# An unusual diverticulum adjacent to two large colonic polyps; a case report

**DOI:** 10.1186/s12876-018-0816-9

**Published:** 2018-06-14

**Authors:** John Schembri, Jonathan Bury, Lesley Hunt, Stuart Riley

**Affiliations:** 1 Department of Gastroenterology, Sheffield Teaching Hospitals, Northern General Hospital, Herries Road, Sheffield, S5 7AU England; 2 Department of Radiology, Sheffield Teaching Hospitals, Northern General Hospital, Herries Road, Sheffield, S5 7AU England; 3 Department of Colorectal Surgery, Sheffield Teaching Hospitals, Northern General Hospital, Herries Road, Sheffield, S5 7AU England

**Keywords:** Ureterosigmoidostomy, Adenoma, Colorectal cancer, Surveillance

## Abstract

**Background:**

Adenocarcinomas can arise in a variety of circumstances in which intestinal segments have been used for urinary diversions. Whereas ureterosigmoidostomy is the oldest and simplest form of continent urinary diversion it also seems to be the most dangerous in this regard. Herein we present a case of colonic neoplasia complicating a non-functioning ureterosigmoidostomy after 55 years; the longest latent period documented to date.

**Case presentation:**

A 56-year-old lady born with congenital bladder exystrophy and who had a functional ileal conduit presented to us with a 6 month history of change in bowel habit and rectal bleeding. Prior to this she had had multiple abdominal surgeries as a child and had suffered from lifelong recurrent urinary tract infections.

Colonoscopy revealed the presence of two large sessile polyps in close proximity to a diverticulum-like structure that after surgical resection turned out to be a non-functioning ureterosigmoidostomy from when she was an infant.

**Conclusions:**

Our case highlights the importance of enrolling patients with ureterosigmoidostomies into long-term colonoscopic surveillance programmes. This is also true for those patients who undergo revisional surgery but have preserved ureteric stumps. Endoscopists should be aware of the varied endoscopic appearances of the anastamosis in order to be able to recognise these structures when present.

## Background

Ureterosigmoidostomy (US) is the oldest and simplest form of continent urinary diversion. Its association with colonic cancer is well established and a 100-fold increased risk of malignancy has been suggested [1]. Characteristically there is a long latent period before the occurrence of cancer and even though most patients subsequently underwent revisional surgery those with intact ureteric stumps also seem to be at risk [2]. Herein, we report a case of colonic malignancy developing adjacent to a non-functioning US initially mistaken for a diverticulum, 55 years after initial surgery.

## Case presentation

A 56-year-old lady presented with a 6 month history of rectal bleeding, passage of mucus and a change in bowel habit to more frequent stools. She had no abdominal pains and her weight was maintained. The patient had been born with bladder exstrophy and had multiple surgeries culminating in a cystectomy with ileal conduit formation at 5 years of age. She had suffered with recurrent urinary tract infections for most of her childhood and adult life but was otherwise well with no other major co-morbidities or risk factors for colorectal malignancy and had no family history of colorectal disease.

**Fig. 1:**
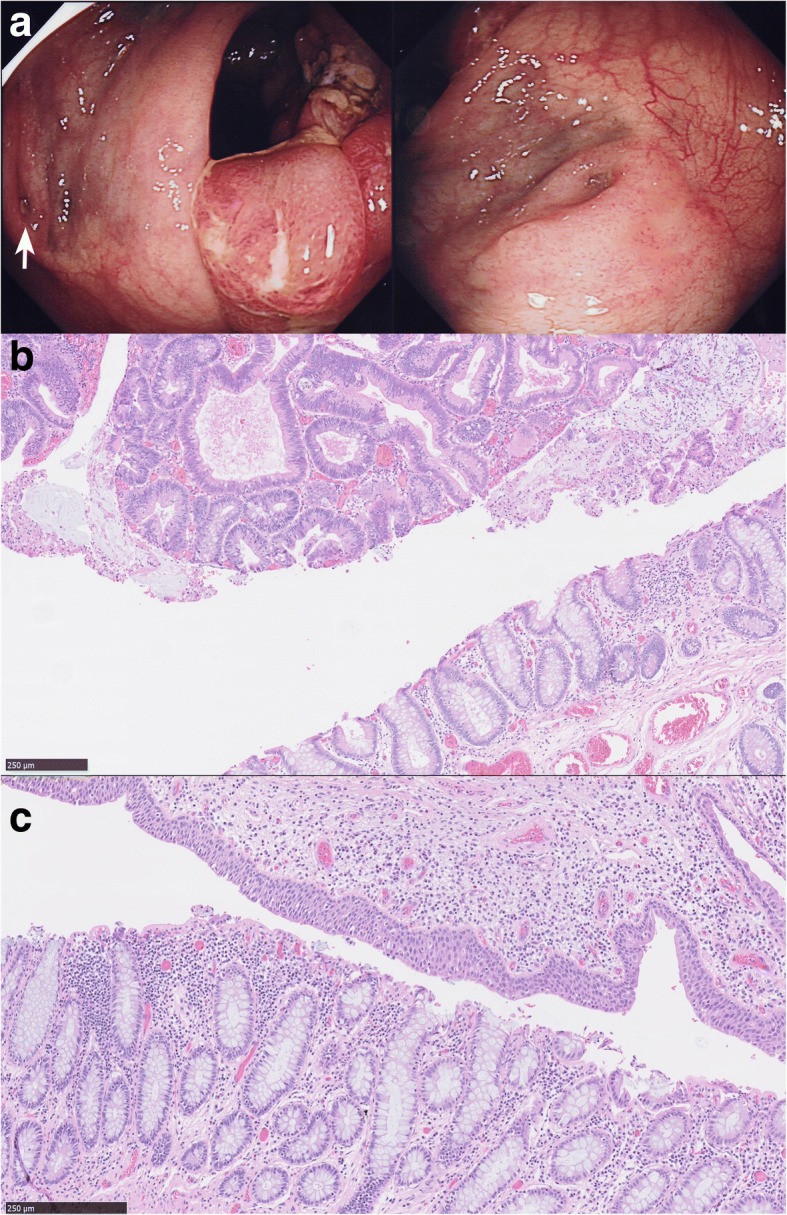
**a** Endoscopic view of diverticulum in close proximity to sessile lesion (white arrow; left panel) and close up of raised diverticular structure (right panel). **b** Magnified view of the H & E-stained histological sections from the sessile lesion showing a typical colonic adenoma with dysplasia. **c** Magnified view of the H & E-stained histological sections from the diverticulum-like structure showing urothelium adjacent to normal colonic mucosa

Colonoscopy revealed two large sessile polyps in the sigmoid colon in close proximity to each other and adjacent to a diverticulum-like structure (Fig. 1a). Each polyp was approximately 3 cm in size and both exhibited a type IV pit pattern with areas of irregularity suggestive of focally advanced disease. Histological examination confirmed both polyps were adenomas comprising both low and high grade dysplasia, without submucosal invasion (Fig. 1b). On closer inspection the mucosa around the diverticulum was also atypical but not adenomatous. The remainder of the colonoscopy was unremarkable with no other evidence of diverticular disease or polyps elsewhere.

Endoscopic resection was considered as a therapeutic option however in view of the above characteristics as well as difficult endoscopic access surgery was preferred. Furthermore, radiological imaging had initially raised the possibility of invasive disease in view of sigmoid thickening. The patient underwent high anterior resection and an open approach was chosen because of suspected intra-abdominal adhesions following extensive pelvic surgery. An end colostomy was formed at the patient’s pre-operative request. At laparotomy the right fallopian tube was adherent to the sigmoid colon and adjacent to this a blind ending tube was noted to emerge from the anti-mesenteric border of the colon. This was marked for pathological identification.

Our patient went on to have an uneventful recovery and her quality of life following surgery was good. Her wish to have a permanent colostomy stemmed from the fact that she had always suffered from an erratic bowel habit and that she was already knowledgeable with regards to stoma care in view of her pre-existing ileal conduit.

## Discussion and conclusions

The blind ending tube was the remnant of a ureteric implant and the diverticulum-like structure was the site of this US (Fig. 1c). Review of the patient’s records revealed that US had preceded ileal conduit formation when she was still an infant. Although adenomas are often sporadic, the absence of a family history and the development of two large adenomas adjacent to the anastomosis suggests a causal link.

Though initially described in US, all forms of urinary diversion have been associated with an increased risk of intestinal malignancy, typically after a long latent period. In US the incidence of colonic carcinoma is between 2 and 15% with an average age of 33 years and a mean interval from the procedure of 26 years. The shortest and longest reported latencies are 3 and 53 years respectively [3, 4]. Whilst no specific risk factors have been linked to the shorter latency tumours, smoking and tobacco-derived urinary carcinogens might play a role in some cases especially if the diversion has been performed subsequent to bladder cancer resection [5].

Malignancy complicating US is nearly always colorectal in origin, does not seem to arise from urothelium and always develops in close proximity to the anastamotic site. The pathogenesis of these tumours is unclear and multifactorial in nature. The most accepted theory suggests that production of nitrite and N-nitroso compounds from nitrate by bacterial flora in the presence of neutral colonic pH is responsible for carcinogenesis [6]. However, experimental data is conflicting since tumour induction has been achieved in rat models irrespective of nitrosamine formation and whilst it has also been suggested that the interaction of both urine and faeces is necessary for carcinogenesis to occur malignancy can develop in bowel segments only exposed to the urinary stream without faecal interaction [7].

Endoscopically the ureterosigmoidoscopy had the superficial appearance of a diverticulum however these sites may also appear as a small cherry-like structure and caution should be exercised not to inadvertently undertake polypectomy thereby disrupting the anastomosis. With careful inspection and modern-day endoscopic imaging this should be easily avoided. Biopsy sampling would clarify if doubt remained. Endoscopic resection of adenomas close to the anastomosis is feasible but care should be taken to ensure the integrity of the US. Intravenous indigocarmine can be used to identify the ureteric orifices.

Whilst US was the mainstay of urinary diversion up to the 1950s many patients ran into problems with hyperchloraemic acidosis and troublesome diarrhoeas, sometimes with faecal incontinence. A growing awareness of the link with colorectal cancer led to the use of ileal conduits as the preferred option, which do not appear to be susceptable to such change. Revisional surgery was undertaken in many but often, as in this case, the ureterosigmoidoscopy site was left in situ. Unfortunately such patients still appear to have the same increased risk of developing sigmoid tumours exhibiting the same latencies, even in cases where exposure to the urinary stream amounted to 6 months or less [2]. Current guidance suggests that if a ureterosigmoidoscopy is converted to another form of urinary diversion then the site of implantation of the ureters into the sigmoid should be excised [8].

The latent period between US and colonic tumours is characteristically long and even though US is no longer the procedure of choice for infants born with bladder exstrophy it is still considered a viable option to preserve continence. Since most of these patients have had the procedure performed in childhood enough time would have passed for neoplasia to develop before they start undergoing bowel cancer screening, despite the long latency. When encountering this group of patients physicians should ensure they are enrolled in long-term annual surveillance programmes [8]. This would have also been true for our patient, who despite having an alternative form of urinary diversion for most of her life, still had intact US sites that had not been excised at the time of revisional surgery.

## Abbreviation

US: Ureterosigmoidostomy
